# Response to perturbation during quiet standing resembles delayed state feedback optimized for performance and robustness

**DOI:** 10.1038/s41598-021-90305-4

**Published:** 2021-05-31

**Authors:** Ambrus Zelei, John Milton, Gabor Stepan, Tamas Insperger

**Affiliations:** 1 MTA-BME Research Group on Dynamics of Machines and Vehicles, Budapest, 1111 Hungary; 2 The Claremont Colleges, W. M. Keck Science Center, Claremont, CA 91711 USA; 3 Department of Applied Mechanics, Budapest University of Technology and Economics, Budapest, 1111 Hungary; 4 MTA-BME Lendület Human Balancing Research Group, Budapest, 1111 Hungary

**Keywords:** Motor control, Sensorimotor processing, Dynamical systems, Biomedical engineering

## Abstract

Postural sway is a result of a complex action–reaction feedback mechanism generated by the interplay between the environment, the sensory perception, the neural system and the musculation. Postural oscillations are complex, possibly even chaotic. Therefore fitting deterministic models on measured time signals is ambiguous. Here we analyse the response to large enough perturbations during quiet standing such that the resulting responses can clearly be distinguished from the local postural sway. Measurements show that typical responses very closely resemble those of a critically damped oscillator. The recovery dynamics are modelled by an inverted pendulum subject to delayed state feedback and is described in the space of the control parameters. We hypothesize that the control gains are tuned such that (H1) the response is at the border of oscillatory and nonoscillatory motion similarly to the critically damped oscillator; (H2) the response is the fastest possible; (H3) the response is a result of a combined optimization of fast response and robustness to sensory perturbations. Parameter fitting shows that H1 and H3 are accepted while H2 is rejected. Thus, the responses of human postural balance to “large” perturbations matches a delayed feedback mechanism that is optimized for a combination of performance and robustness.

## Introduction

For over 50 years, responses to perturbations have been used to investigate the feedback control of human balance^1–3^. These studies established that human balance is not maintained by stereotyped reflexes. Instead, with development, balance control emerges as the nervous system learns to apply generalized rules for maintaining balance^4^. In healthy individuals this ability to adapt and improve balance in a feedback-driven manner does not appear to decline with age^5^. This observation underscores the current development of perturbation-based balance training protocols to improve reactive balance in the elderly as a way to reduce their risk of falling^3,6^.

A large variety of perturbations have been used to disturb human standing balance including sudden platform translations, pulls and tugs^1,2,7–10^. The nervous system responds with a continuum of “ankle” and ”hip” strategies, the exact combination depending on the trade-offs between the required effort and the degree of postural instability to be overcome^11–14^. Despite these observations, theoretically-motivated investigations suggest that common underlying principles may be at work^8,14–17^. Thus, the challenge has become to identify the nature of the governing principles^7,18,19^.

**Figure 1:**
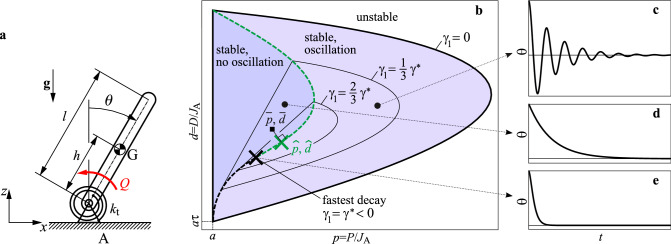
Main concept: dynamic behaviour of balancing by delayed feedback. (**a**) Inverted pendulum model for human standing balance. (**b**) Stability diagram for delayed PD feedback $Q(t)=P \theta (t-\tau ) + D \dot{\theta }(t-\tau )$. Light and dark grey shading indicates oscillatory (spiral type) and nonoscillatory (node type) stable responses, respectively. The two types of responses are separated by the node-spiral separation line indicated by (black-green) dashed line. Contour lines $\gamma _1={\rm const}$ are associated with different settling time of the response. Black $\times$ marker shows the parameter point $(p^*,d^*)$ associated with fastest settling time. Green  marker shows the parameter point $(\hat{p}, \hat{d})$ on the node-spiral separation line that is closest to the experimentally fitted parameter point $(\bar{p}, \bar{d})$. (**c–e**) Responses for different control gains (*p*, *d*). Fastest response is shown in panel (**e**).

The dynamics of human postural sway during quiet standing with eyes closed is very complex and has been described in terms of stochastic and even chaotic motions^20,21^. The underlying mathematical model can be either an inherently stochastic process^22,23^ or a deterministic nonlinear feedback mechanism governed by some kind of intermittent control^24–27^ or their combinations^28–30^. One of the simplest physiological mechanisms for generating a chaotic motion is the interplay between a time-delayed, sampled data system and a sensory dead zone, i.e., corrective actions take place only when the sensory inputs exceed some threshold values^21^. Model based analysis of postural sway is therefore a difficult task since the governing deterministic dynamics may be hidden in the seemingly noisy/chaotic response. Here we employ perturbations in the anteroposterior (AP) and the posteroanterior (PA) directions during quiet standing that are large enough to produce excursions that are significantly larger than the magnitude of the fluctuations in postural sway and the size of sensory dead zones. In this way, the response to the perturbation is not affected by the local noisy/chaotic dynamics and the underlying feedback mechanisms can be identified distinctly by numerical fitting techniques.

Inverted pendulum models are widely used to investigate human balance^19,31–35^ and are currently thought to be “functionally correct”^34,36^. Reactive balance control, that is maintaining balance in the response to a perturbation, is inherently a feedback sensorimotor process in which muscles are activated in direct response to task-level error^14,37–39^. However, the time delayed nature of the feedback control has important implications for the feedback controlled response of balance to perturbations. Our concept is illustrated in Fig. 1. This figure shows the stability diagram for an inverted pendulum stabilized by a time-delayed proportional-derivative (PD) feedback controller. The stable region in the plane (*p*, *d*) of the control gains is characteristically D-shaped^31,40^. For every choice of the control gains located within this D-shaped region, the upright position recovers from a perturbation; however, not all control gains exhibit the same dynamical response to the perturbation. The perturbed responses range from a monotonic exponential recovery to the upright position to recoveries which exhibit an oscillatory component.

The boundary between the monotonic and oscillatory responses is formed by the *node-spiral separation line* (black-green dashed line). The exponential decay of the response is shown by solid contour lines: the smaller the value of $\gamma _1$, the faster the response. Namely, $|\theta (t)| \lessapprox \theta _0 {\rm e}^{\gamma _1 t}$ where $\theta _0$ is the initial angle. $\gamma _1 < 0$ is called exponential decay rate^41^. The fastest responses to perturbations are for choices of the gains in the lower left quadrant of the stability diagram. The optimal point with respect to the response’s settling time is $(p^*,d^*)$ (black $\times$ marker), which is associated with the maximal achievable decay rate $\gamma _1 = \gamma ^*$. This parameter point lies on the node-spiral separation line and divides it into two sections indicated by black and green color. Small changes in *p* or in *d* in the lower (black) branch of the separation line results in significantly larger changes in the decay rate $\gamma _1$ than the same changes do in the upper (green) branch. Hence, the system is more robust to changes in the control gains when operating at a parameter point on the upper (green) branch rather than on the lower (black) one. Note that perturbation of the gains can directly be linked to perturbations in sensory perception since the actual control force is determined as the product of the control gains *p* and *d* and the corresponding sensory inputs, the angle $\theta$ and the angular velocity $\dot{\theta }$. This suggests that the control gains should be selected based on a goal function which takes into account both fast response and robustness. This would give an operation point $(\hat{p}, \hat{d} \,)$ on the upper branch indicated by green  marker.

The main goal of this paper is to compare the dynamics of the time-delayed PD feedback model to measured responses to perturbations during quiet standing. Control gains and feedback delays are estimated by fitting the response of the mechanical model to the measured time histories. The fitted parameter point is indicated by $(\bar{p}, \bar{d})$ in Fig. 1b (black $\blacksquare$ marker). We pose three hypotheses related to the location of the fitted control gains.

1. The fitted control gains are tuned towards the node-spiral separation line, which would indicate that reducing oscillatory response is one of the main goal of the feedback mechanism. This concept can be linked to a critically damped oscillator in the sense that when the proportional gain *p* (which operates as a kind of artificial active stiffness) is fixed, then the derivative gain *d* (a kind of artificial damping) is tuned towards the node-spiral separation line and the resulted motion resembles that of a critically damped oscillator.
2. The fitted control gains are close to the parameter point $(p^*,d^*)$ associated with the fastest possible recovery of balance to perturbations.
3. The fitted control gains are located close to a parameter point $(\hat{p},\hat{d})$ on the upper (green) branch of the node-spiral separation line (i.e., $\hat{p} > p^*$ and $\hat{d} > d^*$) that assures fast recovery even upon small uncertainties in the control gains.

We base our hypotheses on three observations: (1) PD controllers with gains located in the lower left quadrant of the stability diagram are most robust to the effects of random perturbations^42^; (2) expert pole balancers increase maneuverability while minimizing energetic costs for balance control by adapting gains in the lower left quadrant of the stability diagram^43^; and (3) the control gains for subjects who do ball-and-beam balancing are progressively tuned towards the node-spiral separation line as their skill improves^44^. Statistical analysis show that H1 and H3 are accepted and H2 is rejected.

## Results

### Experiments and responses to perturbation

**Figure 2:**
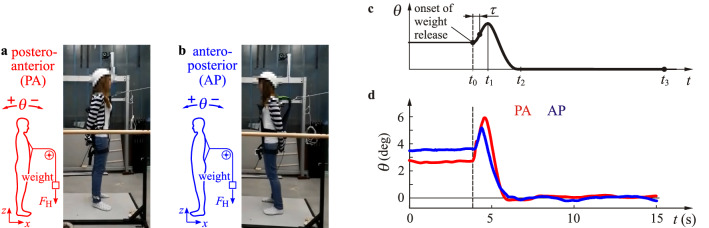
Experimental setup and responses. (**a,b**) Setup for posteroanterior (PA) and anteroposterior (AP) direction. (**c**) Typical time response and some characteristic time instants, $t_0$: onset of perturbation, $t_1$: maximum excursion, $t_2$: recovery to normal posture, $t_3$: end of the trial. (**d**) Sample time signals measured during PA (red) and AP (blue) setups.

The measurement setup, a typical response and measured responses for AP and PA perturbations are shown in Fig. 2. After the abrupt release of the weight at time instant $t_0$, the tilt angle $\theta$ first reaches a maximum then recovers to the angle that corresponds to normal posture. The oscillations before the perturbation ($t<t_0$) and after recovery ($t>t_2$) were significantly smaller (std $0.15^\circ$ in average) than the maximum excursion caused by the perturbation ($3.87^\circ$ in average). Since the force was released at an unexpected instant^45^, the subject corrective action was delayed by the reaction time $\tau$, which, in theory, is the difference between the initial time $t_0$ and the time instant where the response has inflection point, i.e., the angular acceleration $\ddot{\theta }$ changes sign. Responses were typically free of overshoot and resemble the responses of a critically damped oscillator^46,47^. Recorded time signals were used to fit parameters *p*, *d* and $\tau$ in the mechanical model.

### Identified control parameters

**Figure 3:**
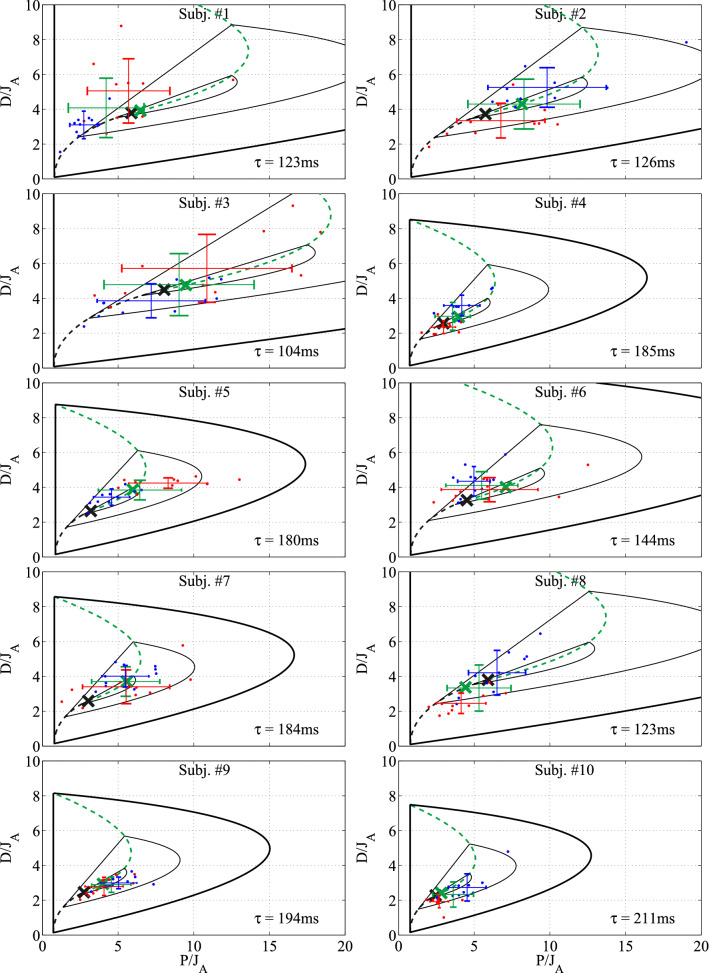
Stability diagrams for the 10 individual subjects and the fitted control parameters. Stability boundary ($\gamma _1=0$) is indicated by thick black line, while thin black lines denote contour curves of $\gamma _1=\frac{1}{3} \gamma ^*$ and $\gamma _1=\frac{2}{3} \gamma ^*$. The node-spiral separation line is shown by black-green dashed line, and the parameter point associated with the fastest decay ($\gamma _1=\gamma ^*$) by black $\times$ marker. Red  markers indicate PA trials, blue  markers indicate AP trials. Means and standard deviations for the PA and the AP trials are shown by red and blue lines, respectively. Green lines indicate the mean and standard deviation for all the 20 trials. The point on the node-spiral separation line that is closest to the mean of the 20 trials is indicated by green  marker.

The control gains *p* and *d* and the time delay $\tau$ were estimated for all the 20 trials (10 PA and 10 AP) for all the 10 subjects. The stability charts together with the identified control gain parameters are shown in Fig. 3 for each subject. The stable region (thick black solid curve) and the node-spiral separation line (dashed black-green curve) correspond to the average delay of the overall 20 trials per subject (the average delay is indicated in each panel). The larger the average delay, the smaller the stable region. All the identified control gains are within the stable region and are distributed close to the node-spiral separation line. Parameter points $(p^*,d^*)$ (fastest decay) and $(\hat{p}, \hat{d} \,)$ (closest point on node-spiral separation line) are indicated by black $\times$ and green  markers for each subject. The basic statistical results of the fitted control parameters $\tau$, *p* and *d* are collected in Table 1. The best fitting time delays were found to be in the range of $100\sim 200$ ms, which is in agreement with different estimates in the literature^47–50^. The identified control gain values also resemble those in the literature that assumes the same delayed PD feedback models of human quiet standing^49,50^.

**Table 1:** Statistics of the estimated reaction time delay $\tau$ and the estimated control gains $p=P/J_{\mathrm {A}}$ and $d=D/J_{\mathrm {A}}$: Mean, Standard Deviation (SD), Median (Med.), Interquartile Range (IQR). Parameters are listed for the trials in the PA and the AP directions separately and together (both).

No.	Mean			SD			Med.			IQR		
	PA	AP	Both	PA	AP	Both	PA	AP	Both	PA	AP	Both
$\tau$ (ms)	169	146	157	93	65	81	165	148	155	140	95	120
$p=P/J_{\mathrm {A}}$ (s$^{-2}$)	5.39	5.36	5.37	3.16	2.51	2.85	4.95	4.70	4.78	3.21	3.52	3.28
$d=D/J_{\mathrm {A}}$ (s$^{-1}$)	3.42	3.69	3.55	1.54	1.19	1.38	3.07	3.62	3.43	1.61	1.62	1.83

### Settling time versus robustness to parameter changes

**Figure 4:**
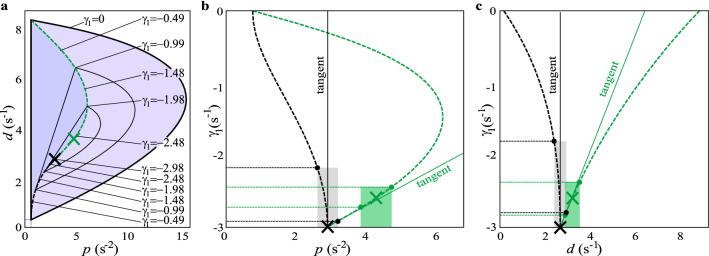
(**a**) Change of the exponential decay rate $\gamma _1$ along the node-spiral separation line. Parameters corresponds to Subject 9. (**b,c**) Dependence of $\gamma _1$ as function of *p* and *d* along the node-spiral separation line. $\pm 10$% perturbation of the gains $(p^*,d^*)$ and $(\hat{p}, \hat{d})$ and the resulted change in $\gamma _1$ is shown by grey and green shading, respectively.

Experiments showed that the fitted control gains are slightly larger than the gains $(p^*,d^*)$ corresponding to the fastest decay. An explanation for this is that the sensitivity of the exponential decay rate $\gamma _1$ to parameter changes is different at different sections of the node-spiral separation line. Note that the response is bounded by $|\theta (t)| \lessapprox \theta _0 {\rm e}^{\gamma _1 t}$, therefore changes in $\gamma _1$ are amplified through an exponential function. The change of $\gamma _1$ along the node-spiral separation line is shown in Fig. 4 for the parameters of Subject 9. Panel a shows the stability diagram with some sample values of $\gamma _1$. It can be seen that $\gamma _1$ changes faster in the lower (black) section of the node-spiral separation line than in the upper (green) one. Panels b and c show the change of $\gamma _1$ as function of *p* and *d*, respectively. The system with control gains selected from the lower branch is more sensitive to changes in *p* and *d* than the system corresponding to the upper (green) branch. Actually, the parameter point $(p^*,d^*)$ is infinitely sensitive to negative changes in *p* and *d*, which can be seen from the vertical slope of its tangent in panels b and c. When applying $\pm 10$% perturbation in the gains $(p^*,d^*)$, then, in the worst case, $\gamma _1$ changes from $\gamma ^*=-2.98$ to $-1.84$. The same $\pm 10$% perturbation on the gains $(\hat{p}, \hat{d})$, in the worst case, results in changes of $\gamma _1$ from $-2.59$ to $-2.38$. Hence, when perturbations/uncertainties are present in the sensory perception, then it is more beneficial to select the control gains form the upper (green) branch of the node-spiral separation line than from the lower (black) one.

Based on this observation, three measures are introduced to describe the response in terms of the control gains. Let the mean of the fitted control gains be denoted by $(\bar{p},\bar{d})$. First, the oscillatory nature of the response related to hypothesis H1 can be described as the distance between the point $(\bar{p},\bar{d})$ and closest point $(\hat{p}, \hat{d})$ of the node-spiral separation line (see Fig. 1). Point $(\hat{p}, \hat{d})$ is defined such that $\sqrt{(\bar{p}-\hat{p})^2+(\bar{d}- \hat{d})^2}$ has to be minimal (for this, the control gains have to be normalized by introducing the dimensionless time $\tilde{t} = t / \tau$). Second, the decay of the response (i.e., settling time) related to hypothesis H2 can be characterized by the distance between the fitted parameter point $(\bar{p},\bar{d})$ and the parameter point $(p^*,d^*)$ associated with the fastest decay. Third, the combined concept of fast decay and robustness to uncertainties in the control gains related to hypothesis H3 can be characterized by the relations $\hat{p} > p^*$ and $\hat{d} > d^*$.

The above discussion implies that the goal function might not be to achieve $(p,d)=(p^*,d^*)$ but to tune the control gains to $(p,d)=(\hat{p}, \hat{d})$, which guaranties a lower limit to $\gamma _1$ even in the case of perturbations of the control gains. This concept minimizes the settling time (e.g., maximizes the magnitude of $\gamma _1$) while at the same time preserves robustness to static perturbations in the control gains (*p*, *d*).

### Hypothesis H1: node-spiral separation line—accepted

Control gains associated with oscillatory and non-oscillatory responses are separated by the node-spiral separation line. Hypothesis H1 can be tested using the distance between the identified mean control gains $(\bar{p}, \bar{d})$ and the closest point $(\hat{p}, \hat{d})$ on the node-spiral separation line. First, the normality of the data was checked by the Anderson-Darling test and it was found that the data are not normally distributed. Therefore, Hypothesis H1 was tested by the non-parametric Wilcoxon signed-rank test (${\mathrm {signrank}}({\bar{p}},{\hat{p}})$ and ${\mathrm {signrank}}({\bar{d}},{\hat{d}})$ in Matlab 2017b), see Table 2. Results show that Hypothesis H1 related to the location of the proportional gain *p* is accepted for most of the subjects (8 out of the 10) and also for the overall data. H1 related to the location of the differential gains *d* is accepted for all the subjects. This observation confirms that the control gains are tuned to be close to the node-spiral separation line.

**Table 2:** Comparison of the experimentally fitted control gains $(\bar{p}, \bar{d})$ to $(\hat{p}, \hat{d})$ (H1) and to $(p^*,d^*)$ (H2) by means of Wilcoxon signed-rank test: rejection of H1 and H2 is indicated by *h* ($h=0$ means that significant difference is not proven statistically, $h=1$ means that there is statistical difference), p-value ($v_p$).

No.	H1:	$\bar{p}$ vs. $\hat{p}$	H1:	$\bar{d}$ vs. $\hat{d}$	H2:	$\bar{p}$ vs. $p^*$	H2:	$\bar{d}$ vs. $d^*$
	*h*	$v_p$	*h*	$v_p$	*h*	$v_p$	*h*	$v_p$
1.	1	0.002	0	0.911	1	0.004	0	0.823
2.	0	0.881	0	0.765	1	0.005	0	0.126
3.	0	0.681	0	0.502	0	0.681	0	0.940
4.	0	0.062	0	0.911	1	0.030	0	0.073
5.	0	0.765	0	0.940	1	0.000	1	0.000
6.	1	0.011	0	0.940	0	0.086	1	0.000
7.	0	0.940	0	0.823	1	0.000	1	0.000
8.	0	0.093	0	0.654	0	0.263	0	0.108
9.	0	0.117	0	0.765	1	0.000	1	0.002
10.	0	0.062	0	0.156	1	0.001	0	0.627
all	0	0.391	0	0.852	1	0.003	1	0.030

### Hypothesis H2: fastest decay of the response—rejected

Statistical analysis was performed in order to check whether the fitted control gain pairs $(\bar{p}, \bar{d})$ correspond to the point $(p^*,d^*)$ associated with the fastest decay. The results of Wilcoxon signed-rank test (${\mathrm {signrank}}({\bar{p}},p^*)$ and ${\mathrm {signrank}}({\bar{d}},d^*)$) are shown in Table 2. In case of most of the subjects and also in case of the overall data, Hypothesis H2 was rejected. Note that this is a weak rejection, since 7 out of the 10 subjects was rejected for *p* and only 4 out of the 10 for *d*. This analysis shows that although the fitted control gain pairs seem to be distributed close to the control gain pairs that yields the fastest decay (i.e., close to the by black $\times$ markers in Fig. 3), this hypothesis is not verified statistically. An explanation to this observation is that the parameter point $(p^*,d^*)$ is infinitely sensitive to small changes in the control gains, hence to the small changes in the perceived sensory feedback.

### Hypothesis H3: fast decay of the response with robustness—accepted

The fitted control gains was shown to be close to the point $(\hat{p}, \hat{d})$ on the node-spiral separation line in H1. Now it is to be checked whether $(\hat{p}, \hat{d})$ lies on the upper or on the lower branch of the separation line. For 9 out of the 10 subjects, it was observed that $\hat{p} > p^*$ and $\hat{d} > d^*$, hence $(\hat{p}, \hat{d})$ lies on the upper (green) branch. This confirms that the control gains are indeed tuned to the more robust section of the node-spiral separation line, hence H3 is accepted. Therefore, it is a plausible assumption that the control gains are tuned to achieve fast decay ($\gamma$-stability) but at the same time allow some variations in the gains or in the sensory feedback.

### No difference between PA and AP parameters

The difference between the fitted control gains obtained for the PA and the AP trials was analyzed using Wilcoxon signed-rank test. No significant difference was found (the p-value for gain *p* was $v_p=0.922$, for gain *d* it was $v_p=0.557$ and for the delay $\tau$ it was $v_p=0.334$). Hence, both AP and PA balancing process can be modeled by delayed feedback with control gains tuned close to $(\hat{p}, \hat{d})$.

### Variation of the fitted gains over the trials—no learning

**Figure 5:**
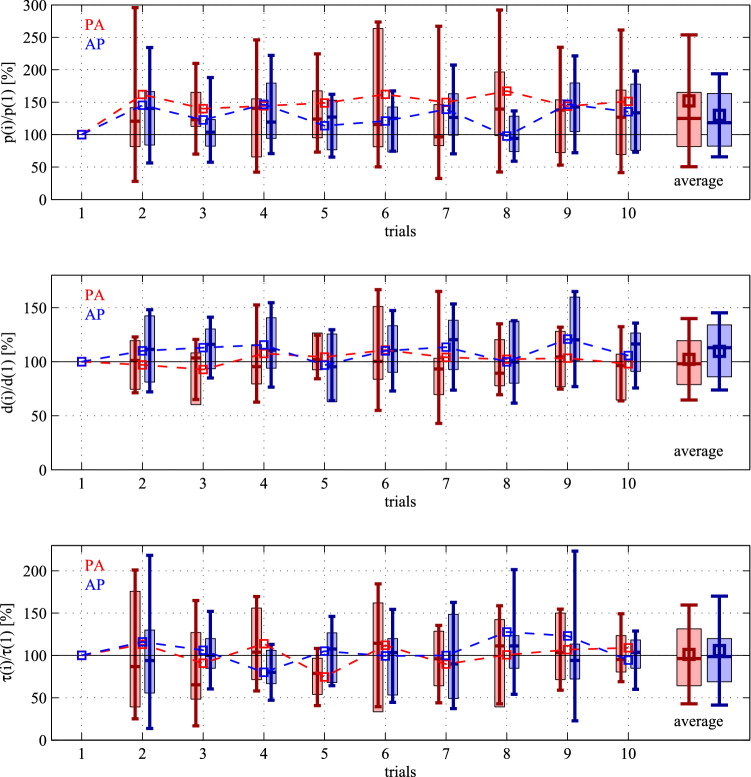
Statistics of the relative variation of the fitted control gains and the reaction delay during the trials compared to the first trial. Square marker and errorbar: mean ± SD for the 10 subjects; boxplot: median and IQR.

The variation of the fitted control gains over the 10-trial series is shown in Fig. 5 in order to check whether the control gains are coherently tuned towards certain region of the plane (*p*, *d*). There is no clear trend in the change of the fitted parameters (either in the mean or in the variations), which suggests that learning process was not present during the trials. Hence, reacting to perturbation during standing still can be considered as an already learned and acquired feedback mechanism.

### Effect of passive stiffness

The above results have been obtained for the mechanical model where the passive ankle stiffness was $k_{{\rm t}}=0.91 mgh$^58^. In order to check the validity of the results, the same calculations and the same parameter estimations were performed for $k_{{\rm t}}=0.67mgh$ too, which is in the lower region of the physiologically plausible stiffness values^59^. Wilcoxon signed-rank test shows that Hypothesis H1 is accepted for the overall data for both the proportional control gains *p* ($h=0$, $v_p=0.478$) and the derivative gain *d* ($h=0$, $v_p= 0.970$), while Hypothesis H2 is rejected for *p* ($h=1$, $v_p= 0.008$) and is weakly accepted for *d* ($h=0$, $v_p=0.067$). Thus, the main results regarding the location of the fitted control gains does not change significantly with the value of the passive ankle stiffness.

## Discussion

The results confirm that recovery of quiet standing after a sudden perturbation can well be described by a delayed state feedback mechanism described by three parameters: the reaction delay $\tau$, the proportional and the derivative control gains *p* and *d*, respectively (see Methods for validation). While the reaction delay is an inherent feature of the control process, the control gains can be tuned to improve performance, namely, to reduce oscillations and settling time. Parameter fitting shows that the control gains are tuned such that the response is non-oscillatory (H1) and result in fast recovery even in case of parameter uncertainties related to the perception of the angular position and the angular velocity (H3). Hence, the typical response is fast and non-oscillatory that resembles the dynamics of a critically damped nondelayed mechanical system^46,51^. This feature of the response is also in agreement with delayed models of human standing in the sense that the control gains are located in the lower left regions of the stability region^42,47,52^.

No significant changes were observed on the parameters over the trials implying that the identified feedback mechanism has been already learned and practiced before during the activities of daily living. The effect of learning process is more pronounced when a new and unknown task is to be performed, e.g, ball-and-beam balancing^44^, balancing on balance board^53^, beam walking^54^ or combined quiet standing and stick balancing^27^. A question arises whether practice or other techniques can be developed to further improve the performance against sudden perturbations.

It shall be mentioned that other than delayed PD feedback is also a possible concept to describe the stabilization process. Several ideas are available in the literature, such as intermittent feedback where intermittency can be either a control logic utilizing the structure of stable and unstable manifolds in the phase plane of the inverted pendulum^24–27,55^ or an inherent consequence of the uncertainties in the operation of the sensory system, e.g., sensory dead zones^19,29^. Further possible control concepts are acceleration feedback^32,56^, predictor feedback^39,43^ or an event-driven combination of ankle and ankle–hip strategies^25^, just to mention a few. An advantage of the delayed PD feedback model is that while it is widely used in the literature^26,31,33,47,50^, it accounts with the two most important features of neural motor control: (1) actuation is performed based on perceived sensory signals; and (2) there is a reaction time delay between sensory perception and action.

The postural responses to anterior (AP) and posterior (PA) perturbations are very complex^1,4,61,62^. For AP perturbations the force generated by contraction of the calf muscles is resisted by the reactant force generated by the standing surface. Presumably this may help to offset the decrease in passive ankle stiffness as the dorsiflexion angle increases^58–61^. In contrast, for PA perturbations the contraction of the calf muscles is not opposed by the surface reactant force. Thus in order to maintain balance coactivation of the anterior and posterior shank muscles becomes important and the restoration of balance depends more on the movements of the trunk^62^. This may explain why the angle $\theta$ is slightly larger for PA than AP responses (see the peaks in Fig. 2d).

We have shown that when the tilt angle ($\approx 3^\circ$) is much larger that the proprioceptive sensory dead zone ($\approx 0.05^{\circ } - 0.08^{\circ }$), the feedback control of the responses to AP and PA perturbations are well described by a simple PD feedback control mechanism for the stabilization of an inverted pendulum. The surprising observation is that the values of the feedback gains are the same for both responses. Thus, as pointed out previously in a different context^34^, modeling human balance using an inverted pendulum works quite well. It may be possible to understand the basis for this observation by using more complicated models for balance control^24,25^. However, it is more likely that the responses to larger perturbations are more relevant for understanding the etiology of falls than investigations into the subtle nature of the fluctuations in balance that occur during quiet standing. Thus, we anticipate that the observation that human balance control responses to large perturbations resembles that of a delayed state feedback optimized for performance and robustness will greatly simplify investigations into the nature of human falls.

## Methods

### Mathematical model

An inverted pendulum model for human standing balance is shown in Fig. 1a. Briefly the human body is modeled as a homogeneous rod of mass, *m*, pivoted on a joint A^30,32^. The equation of motion takes the form

1 $$\begin{aligned} J_{{\rm A}} \ddot{\theta }(t) + k_{{\rm t}} \theta (t) - mgh \sin \theta (t)= -Q(t) \end{aligned}$$

where *h* denotes the distance between the center of gravity and the ankle joint *A*, *g* is the acceleration due to gravity, $J_{{\rm A}}$ is the moment of inertia with respect to point A, and $\theta$ is the general coordinate which describes the angular position of the body with respect to vertical. There are two types of torques which interact to stabilize the upright position. First, there is the passive stiffness of the ankle related to the mechanical properties of the foot, Achilles tendon and aponeurosis. The contribution of these forces to balance is modeled by a torsional spring of stiffness $k_{{\rm t}}$^19,21,30^.

The intrinsic mechanical stiffness of the ankle is not sufficient to maintain stability during quiet standing and contractions of parallel connected calf muscles are required. Thus active muscle contractions produce feedback-driven torques, *Q*(*t*), which act across the ankle joints. The proportional-derivative feedback control had the form

2 $$\begin{aligned} Q(t) = P \theta (t-\tau ) + D \dot{\theta }(t-\tau ) , \end{aligned}$$

where $\tau$ is the reaction time delay, and *P* and *D* are, respectively, the proportional and derivative gains. Substitution into (1) and linearization about the $\theta =0$ upright vertical position gives the delay-differential equation

3 $$\begin{aligned} \ddot{\theta }(t) -a \theta (t) = -p\theta (t-\tau ) - d\dot{\theta }(t-\tau ) \end{aligned}$$

where

4 $$\begin{aligned} a=\frac{(mgh-k_{{\rm t}})}{J_{{\rm A}}} > 0 \end{aligned}$$

is a system parameter and

5 $$\begin{aligned} p=\frac{P}{J_{{\rm A}}}, \qquad d=\frac{D}{J_{{\rm A}}} \end{aligned}$$

are normalized control gains.

The stability of (3) can be determined using the D-subdvision method^40^. Substitution of the exponential trial solution $\theta (t)= B \mathrm {e}^{\lambda t}$ into (3) gives the characteristic function

6 $$\begin{aligned} D(\lambda ) = \lambda ^2 - a+p\mathrm {e}^{-\lambda \tau } + d \lambda \mathrm {e}^{-\lambda \tau }. \end{aligned}$$

The characteristic equation $D(\lambda ) = 0$ has infinitely many complex roots, $\lambda _i$ ($i=1,2,\dotsc ,\infty$), which are called characteristic exponents. The system is stable, i.e, the solution $\theta (t)$ converges to 0, if ${\rm Re}(\lambda _i) < 0$ for all *i*. Taking $\lambda = \gamma \pm i \omega$ and setting $\gamma =0$, the equation $D(\lambda ) = 0$ gives the transition curves

$$\begin{aligned} \text{ if } \omega =0&:&p=a, \quad d \in R, \\ \text{ if } \omega \ne 0&:&p(\omega )=(\omega ^2+a)\cos (\omega \tau ), \quad d(\omega )=\frac{\omega ^2+a}{\omega } \sin (\omega \tau ) \end{aligned}$$

These parametric curves delimit the D-shaped stability region shown in Fig. 1b.

In order to characterize the response associated with different parameter pairs (*p*, *d*), the general solution

7 $$\begin{aligned} \theta (t) = \sum _{i=1}^{\infty } B_i \; {\rm e}^{\lambda _i t} = \sum _{i=1}^{\infty } B_i \; {\rm e}^{{\rm Re}(\lambda _i t)} \Big ( \cos ({\rm Im} (\lambda _i t)) + {\rm i} \sin ({\rm Im} (\lambda _i t)) \Big ) \end{aligned}$$

has to be analysed, where $B_i$ is the complex amplitude corresponding to $\lambda _i$. The values of $B_i$’s are determined by the initial functions (initial perturbations) during $t\in [-\tau ,0]$, but the stability is independent of these parameters. It is assumed that the characteristic exponents are ordered such that ${\rm Re}(\lambda _1) \ge {\rm Re}(\lambda _2) \ge {\rm Re}(\lambda _3) \ge \dots$. The dynamics of the response is determined by the dominant (rightmost) characteristic exponent $\lambda _1$ . While the real part ${\rm Re}(\lambda _1)$ corresponds to the decay of the response (settling time), the imaginary part ${\rm Im}(\lambda _1)$ gives the oscillation frequency. In mathematical terminology, $\gamma _1={\rm Re}(\lambda _1)<0$ is called exponential decay rate and the system is said to be $\gamma$-stable if $\gamma _1 \le \gamma <0$^41^.

Figure 1 shows the dynamic behaviour of (3). The stable parameter region (where $\gamma _1<0$) is bounded by black thick curve. Control gains out of the stable region results in either increasing oscillations or exponential growth, hence, in both cases, falling. Within the stable region, thin curves represent different contour lines of $\gamma$-stability. The larger the magnitude of $\gamma _1$ the shorter the settling time. The fastest response is obtained when $(p,d)=(p^*,d^*)$. Thus, $p^*$ and $d^*$ can be considered as optimal gains with respect to settling time. This concept can be associated to the terminology of critical damping of nondelayed models, which plays an important role in modelling the response to sudden perturbations during standing still^46,47,51^.

The stable region can be separated into two parts based on the imaginary part of the dominant root $\lambda _1$. Darker shaded region to the left from the black-green dashed line is associated with ${\rm Im}(\lambda _1) = 0$. In this case, the dominant solution component $B_1{\rm e}^{\lambda _1 t}$ is non-oscillatory. Lighter shaded region to the right of the dashed line is associated with ${\rm Im}(\lambda _1) > 0$ and the corresponding solution component reads $B_1{\rm e}^{\lambda _1 t} + \bar{B}_1{\rm e}^{\bar{\lambda }_1 t}$, which is oscillatory with angular frequency ${\rm Im}(\lambda _1)$ (here $\bar{\lambda }_1$ and $\bar{B}_1$ are the complex conjugate of $\lambda _1$ and $B_1$, respectively). The black-green dashed line is called *node-spiral separation line* since it separates node type and spiral type solutions^44^. Note that the point $(p^*,d^*)$ corresponding to the fastest response lies on the node-spiral separation line. The node-spiral separation line can be divided into two parts based on the location of the dominant (rightmost) roots. In the lower branch (black dashed line) in Fig. 1b, the rightmost characteristic root is real and has a multiplicity of 2. In the upper branch (green dashed section), a real and a complex pair of characteristic roots coexists with the same real part. At parameter point $(p^*,d^*)$, the rightmost root is real ($\lambda _1=\gamma _1^*$) and has a multiplicity of 3.

Besides the actual values of the exponential decay rate $\gamma _1$, its robustness to changes in the control parameters is also an important feature of the control process. $\varepsilon _{{\rm p}}$ relative error in *p* and $\varepsilon _{{\rm d}}$ relative error in *d* alter the control force as

8 $$\begin{aligned} Q_{{\rm perturbed}}(t) = J_{{\rm A}} \, p (1 + \varepsilon _{{\rm p}}) \theta (t-\tau ) + J_{{\rm A}} \, d (1 + \varepsilon _{{\rm d}}) \dot{\theta }(t-\tau ) , \end{aligned}$$

hence this perturbation can also be implemented as perturbation in the sensory perception of $\theta$ and $\dot{\theta }$ with the same relative error $\varepsilon _{{\rm p}}$ and $\varepsilon _{{\rm d}}$, while the gains *p* and *d* are constant. This suggest a sensitivity analysis of $\gamma _1$ to changes in *p* and *d*.

### Participants

We carried out the experiments with 10 subjects (8 males, 2 females) whose parameters and related statistical data are shown in Table 3. The subjects had no self-reported medical conditions which could affect their ability to perform the required tasks. The research was carried out in accordance with relevant guidelines and regulations following the principles of the Declaration of Helsinki. All subjects provided written informed consent for the procedures, signed a General Data Protection Regulation (GDPR) form and were given the opportunity to withdraw from the study at any time. The research project and the study protocol was approved by the Faculty of Mechanical Engineering, Budapest University of Technology and Economics.

**Table 3:** Parameters of the subjects.

No.	Body mass [kg]	Body height [cm]	Age [years]	Applied force PA [N]	Applied force AP [N]
1.	77	176	33	52	56
2.	57	165	25	33	42
3.	62	184	22	42	42
4.	78	187	21	42	42
5.	50	161	22	27	27
6.	70	173	35	56	56
7.	76	176	42	42	42
8.	83	178	25	42	42
9.	95	192	36	56	56
10.	60	180	22	27	42
min	50	161	21	27	27
max	95	192	42	56	56
Mean	70.8	177	28.3	41.9	44.7
SD	13.6	9.44	7.51	10.6	9.1
Med	73	184	25	42	42

### Procedure

The concept of the measurements is shown in Fig. 2. We perturbed standing balance by the unexpected release of a resisting force^7^. While standing comfortably the subject resists a horizontal force, $F_{{\rm H}}$ provided by hanging a weight via a rope that was connected to the subject by a body harness. A constant force $F_{\rm {H}}$ was applied either in the PA or in the AP direction (Fig. 2a,b, respectively). Under these conditions the subject’s preferred standing position was slightly tilted in order to resist the applied force. The force was released manually using a bolt mechanism at an unexpected moment. This causes an initial sway in the direction opposite to the released force as shown by the peaks in Fig. 2c, d. After some transients, subjects found their new vertical equilibrium (normal posture) and they kept on standing in this position for $t_{{\rm s}}=15$ s. Subjects were instructed to keep their hip and knee joints in a constant extended position and to recover their balance without flexing their knees or hips or moving their arms. The applied force $F_{{\rm H}}$ shown in Table 3 was the largest one that the subject was able to resist without any difficulties. Each subject performed 10 trials with $F_{{\rm H}}$ applied in one direction (either PA or AP) followed by 10 trials in which $F_{{\rm H}}$ was applied in the other direction. In order to prevent carry order effects, the direction of $F_{{\rm H}}$ for the first 10 trials was chosen randomly.

The time instant when the weight was released is $t_0$. Maximum excursion was reached at time instant $t=t_1$, normal posture was recovered at $t=t_2$, after that subjects kept on standing quietly for $t_{{\rm s}}=t_3-t_2=15$ s and the balancing trial was terminated after time instant $t=t_3$. Response was evaluated and parameter fitting was performed over the period $t \in [t_1,t_1 + t_{{\rm p}}]$ with $t_{{\rm p}}=10$ s. The time signal over the period $t \in [t_2,t_3]$ was used to calibrate the normal posture such that the mean value of the tilt angle $\theta (t)$ during this period was zero.

### Data collection

A high-speed motion capture system (8 synchronized OptiTrack Prime13 cameras, 120 Hz) was used to measure the three dimensional position $(x_i(t), y_i(t), z_i(t))$ of spherical reflective markers (diameter $16\,\mathrm {mm}$) where the subscript *i* refers to the location of the markers: $i=0,1,2,3,4$ respectively stand for the ankle, knee, hip, shoulder, and head. Projecting all movements to the anterior-posterior plane (*x*, *z*), the absolute tilt angles can be calculated as

9 $$\begin{aligned} \theta _i = \arctan \left( \frac{x_i(t)-x_0(t)}{z_i(t)-z_0(t)} \right) , \end{aligned}$$

where $i=1,2,3,4$ respectively indicate ankle–knee, ankle–hip, ankle–shoulder and ankle–head angles.

### Validity of the single inverted pendulum model

**Figure 6:**
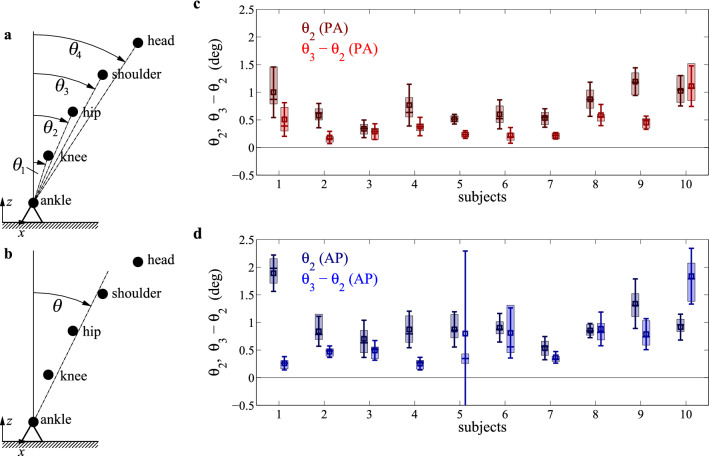
Measured tilt angles. (**a**) Absolute tilt angles: ankle–knee ($\theta _1$), ankle–hip ($\theta _2$), ankle–shoulder ($\theta _3$) and ankle–head ($\theta _4$) angles. (**b**) Average tilt angle of the body used for the single inverted pendulum model. (**c,d**) Average and variations of the RMS values of the angles across the ankle ($\sim \theta _2$) and the hip ($\sim \theta _3-\theta _2$) for the individual trials per subjects for PA (**c**) and AP (**d**) perturbations.

Tilt angles $\theta _i$ ($i=1,2,3,4$, ankle–knee, ankle–hip, ankle–shoulder and ankle–head angles) were calculated based on the marker positions located on the ankle, knee, hip, shoulder and head, respectively. Measurements show that most of the corrective motion happen at the ankle joint as the participants were instructed to keep the knee and hip joints fixed. The angle across the knee joint was found to be small, i.e., $\theta _1 - \theta _2 \approx 0$, and was further reduced at perturbation onset by contraction of the rectus femoris muscle^45^. Hence, $\theta _2 \approx \theta _1$ provides a good measure of the ankle across the ankle joint. The distribution of ankle–hip angle $\theta _2$ and the difference between the ankle–shoulder and the ankle–hip angles ($\theta _3-\theta _1$) are shown in Fig. 6. In average, about $75\%$ of the corrective motion takes place at the ankle joint and only $\sim 25\%$ at the hip joint. Correlation between the angle across the ankle joint ($\theta _2$) and the ankle–hip angle ($\theta _3$) for all subjects were above 0.9, which reflects that corrections at the ankle and hip joints are performed in phase. These observations suggest that a single inverted pendulum model can be used to capture the main characteristics of postural sway^30,33,34,50^. The tilt angle associated with the single pendulum model of the body was calculated as the average of the ankle–hip, ankle–shoulder and ankle–head angles:

10 $$\begin{aligned} \theta (t) = \frac{1}{3} \left( \theta _2(t) +\theta _3(t) + \theta _4(t) \right) . \end{aligned}$$

### Parameters for the mechanical model

The dynamic parameters for the mechanical model was estimated using anthropometric data based on the mass and the height of the subjects. The mass moment of inertia with respect to the ankle joint was calculated as $J_{{\rm A}} = J_{{\rm G}} + m h^2$ where $J_{{\rm G}}= \frac{1}{12} \nu m \ell ^2$, $\ell$ is the total body height and $\nu = 0.6$^57^.

Estimations for the passive ankle stiffness ratio $k_{{\rm t}}/mgh$ ranges between 0.44 and 0.91 and it is typically smaller for larger rotations^58–61^. For the calculations, we set an upper estimate $k_{{\rm t}}=0.91mgh$^58^ and the validity of the results is also checked for a lower estimate $k_{{\rm t}}=0.67mgh$^59^.

### Parameter estimation

The control parameters *p*, *d* and the reaction delay $\tau$ were estimated using a cost function constructed as the integral of the residual of the model equation (3)  namely,

11 $$\begin{aligned} R(p,d,\tau ) = \int \displaylimits _{t_1}^{t_1+t_{\mathrm {p}}} \left( \ddot{\theta }(t) -a \,\theta (t) + p\,\theta (t-\tau ) + d\,\dot{\theta }(t-\tau ) \right) ^2 \mathrm {d}t. \, \end{aligned}$$

The estimated control parameters were assessed by minimizing the residual *R*. First, *R* was calculated for a series of fixed $\tau$ values over the interval $\tau \in [0, 0.4]\,\mathrm {s}$ with resolution $\Delta \tau _1 = 0.025$ s and the best fitting parameters *p* and *d* were determined as a result of a linear algebraic problem. Then $\tau$ was swept on a refined grid with $\Delta \tau _2 = 0.005$ s in the vicinity of the previously best fitting $\tau$ value. The reason for such a two-step parameter estimation was that minimization of *R* with respect to *p*, *d* and $\tau$ at the same time requires a nonlinear searching algorithm, while minimization with respect to *p* and *d* for a fixed delay $\tau$ gives a linear problem. Furthermore, the value of the reaction delay is bounded between $\sim 100$ and $\sim 200$ ms^47–50^, while the values of the control gains may vary to a larger extent. The result of the fitted control gains and the average reaction delay are shown in Fig. 3.
